# Sensory neuron cultures derived from adult db/db mice as a simplified model to study type-2 diabetes-associated axonal regeneration defects

**DOI:** 10.1242/dmm.046334

**Published:** 2021-01-22

**Authors:** Cristian De Gregorio, Fernando Ezquer

**Affiliations:** Center for Regenerative Medicine, School of Medicine Clínica Alemana-Universidad del Desarrollo, Santiago, 7690000 Chile

**Keywords:** Type-2 diabetes mellitus, Diabetic neuropathy, Adult sensory neuron cultures, Neuritic regeneration, Dorsal root ganglia

## Abstract

Diabetic neuropathy (DN) is an early common complication of diabetes mellitus (DM), leading to chronic pain, sensory loss and muscle atrophy. Owing to its multifactorial etiology, neuron *in vitro* cultures have been proposed as simplified systems for DN studies. However, the most used models currently available do not recreate the chronic and systemic damage suffered by peripheral neurons of type-2 DM (T2DM) individuals. Here, we cultured neurons derived from dorsal root ganglia from 6-month-old diabetic db/db-mice, and evaluated their morphology by the Sholl method as an easy-to-analyze readout of neuronal function. We showed that neurons obtained from diabetic mice exhibited neuritic regeneration defects in basal culture conditions, compared to neurons from non-diabetic mice. Next, we evaluated the morphological response to common neuritogenic factors, including nerve growth factor NGF and Laminin-1 (also called Laminin-111). Neurons derived from diabetic mice exhibited reduced regenerative responses to these factors compared to neurons from non-diabetic mice. Finally, we analyzed the neuronal response to a putative DN therapy based on the secretome of mesenchymal stem cells (MSC). Neurons from diabetic mice treated with the MSC secretome displayed a significant improvement in neuritic regeneration, but still reduced when compared to neurons derived from non-diabetic mice. This *in vitro* model recapitulates many alterations observed in sensory neurons of T2DM individuals, suggesting the possibility of studying neuronal functions without the need of adding additional toxic factors to culture plates. This model may be useful for evaluating intrinsic neuronal responses in a cell-autonomous manner, and as a throughput screening for the pre-evaluation of new therapies for DN.

## Abstract (precis)

**Summary:** Morphological characterization of a model for evaluating neuritic regeneration *in vitro* in dorsal root ganglion primary neurons derived from type-2 diabetic mice with an advanced stage of diabetic neuropathy.

## INTRODUCTION

Diabetes mellitus (DM) is a multifactorial chronic disease characterized by a sustained hyperglycemic status. According to the International Diabetes Federation, 463 million people worldwide suffer from DM. Type-2 diabetes (T2DM), which is the most common type of diabetes, represents over 90% of total diabetes cases (International Diabetes Federation, 2019). DM has also been associated with several clinical complications affecting the heart, kidney, retina, vascular system and nervous system (Forbes and Cooper, 2013).

Diabetic neuropathy (DN) is the earliest and most prevalent chronic complication of diabetes, affecting up to 50% of DM patients (Argoff et al., 2006). Distal symmetric polyneuropathy is the most common form of DN (which normally occurs in a ‘stocking and glove’ distribution) and is proposed to reflect the dying-back of the longest peripheral nerve fibers in upper and lower limbs (Feldman et al., 2019). Patients afflicted with DN perceive a significant reduction in life quality due to sensitivity loss, chronic pain and muscle atrophy (Vincent et al., 2004), which are associated with a high risk of developing diabetic foot ulcerations and limb amputation (Volmer-Thole and Lobmann, 2016).

As DN has no available cure, the current management has been focused on improving glycemia control and promoting a healthy lifestyle. However, this is extremely difficult to achieve, as most diabetic patients do not follow the treatments. Additionally, strict glucose control seems to have only a partial effect in reducing DN progression in T2DM patients (Feldman et al., 2019). Neuropathic pain is normally treated with palliative drugs, such as anti-epileptics, antidepressants and opioids, but side effects mean they are not recommended as long-term treatments (Boulton et al., 2005). The highly complex and multifactorial etiology of DN has hindered the generation of comprehensive treatments. The current experimental strategies to treat or prevent DN aim to block the mechanisms involved in its development, including inhibition of several metabolic pathways, control of oxidative stress, reduction of chronic inflammation, stimulation of vasculogenesis and administration of neuroprotective factors (Pittenger and Vinik, 2003; Schratzberger et al., 2001; Shi et al., 2013a,b; Zhou and Zhou, 2014). Even though these treatments have generated promising results in experimental models of DM, none of these therapies have been effective when translated at the clinical level, suggesting the necessity for developing new models and identifying new therapeutic targets for DN (Muller et al., 2008).

Different animal models of DM have been used to study the pathogenesis of DN; for example, streptozotocin-induced or genetic-induced diabetic rodents on normal or high-calorie dietary regimes (O'brien et al., 2014; Reuter, 2007; Sullivan et al., 2007). However, the complexity of *in vivo* models has inherent limitations for studying molecular events and signal transduction pathways. Alternative *in vitro* model systems enable the evaluation of the relative contribution of specific cell types in the peripheral nervous system (PNS) to DN pathogenesis, in a controlled extracellular environment (Sango et al., 2006). Furthermore, *in vitro* cultures may be useful for throughput assays for the pre-screening of new potential therapeutic molecules that could prevent or reduce morphological, functional, or electrophysiological defects associated with hyperglycemia exposition (Melli and Höke, 2009; Vincent and Feldman, 2007). Additionally, *in vitro* cultures offer the advantage of reducing the number of animals needed, as the tissue obtained from one animal could be used to evaluate many different experimental conditions.

Several studies regarding the culture of neonatal rodent cells have shown that high glucose levels promote neuronal reactive oxygen species (ROS) production, mitochondrial dysfunction, neurite outgrowth defects and cell death in dorsal root ganglion (DRG) sensory neurons (Russell et al., 2002; Vincent et al., 2005; Singh et al., 2017; Oses et al., 2017). Furthermore, hyperglycemia has been shown to affect Schwann cell (SC) proliferation, migration and SC-neuron communication, which constitute crucial events during axonal regeneration (Gumy et al., 2008; Tosaki et al., 2008). Other studies have been based on dissociated DRG sensory neurons derived from adult diabetic animals, which offer the possibility of studying completely developed neurons. Studying neurons from adult animals is advantageous because they are not dependent on neurotrophins or serum for survival; so, they can be cultured in a defined medium of interest (Melli and Höke, 2009). To date, the vast majority of *in vitro* studies with adult primary neuronal cultures have been derived from diabetic animals treated with chemical agents that destroy pancreatic β-cells (such as streptozotocin or alloxan), which does not reliably represent the clinical signs of T2DM (reviewed by Sango et al., 2006). In addition, these experimental models present phenotypes that are usually milder and less comparable to human disease than what occurs with the BKS db/db mouse, probably the most robust model for DN described so far (Sullivan et al., 2007). Unlike T2DM, type-1 DM (T1DM)-associated DN progression is highly dependent on glucose control, suggesting that pathological mechanisms leading to DN progression in T2DM go beyond just hyperglycemia. Obesity and chronic hypertension, dyslipidemia, inflammation and insulin resistance are important contributors to DN in T2DM patients, thus, an acute *in vitro* or *in vivo* hyperglycemic state could not represent the mechanisms triggering axonal degeneration and cell apoptosis in this disease (Callaghan et al., 2012; De Gregorio et al., 2018). We propose that neuronal cultures derived from aged T2DM animals could represent, in a more accurate way, the environmental chronic damage suffered by a specific cell type or tissue.

To our knowledge, there are currently no well-characterized *in vitro* models to study neuronal function in advanced stages of DN derived from T2DM. Several studies have reported the culturing of adult DRG neurons in spontaneous T2DM adult mice; one of these studies used 8-week-old *ob/ob* mice to analyze the morphological response of neurons derived from diabetic animals to insulin (Grote et al., 2011); other studies have used DRG neurons derived from adult *db/db* mice to evaluate mitochondrial function and Ca^+2^ homeostasis *in vitro* (Kostyuk et al., 1999; Ma et al., 2014; Vincent et al., 2010). However, in these cases, neuronal cultures were not morphologically characterized in basal culture conditions; thus, it is not possible to determine the consequences of chronic exposition to a noxious environment over the neuritic regeneration capacity in these sensory neuron cultures *in vitro*.

In this study, we established an optimized protocol to cultivate DRG neurons derived from 6-month-old BKS db/db mice, a robust T2DM model that exhibits an advanced progression of DN at this age (De Gregorio et al., 2018). We used the ability of the DRG neuron to regenerate their neurites as an easy-to-analyze readout of neuronal function, and then we evaluated their capability to respond to commonly used neuritogenic molecules. Finally, as a proof of concept, we used this model to study a potential therapy for DN based on the mesenchymal stem cells (MSC) secretome, which has showed promising therapeutic effects in previous trials in embryonic DRG neurons subjected to hyperglycemia (Oses et al., 2017), and in an animal model of T2DM (De Gregorio et al., 2020).

## RESULTS

### Diabetic *db/db* mice as a model for T2DM

Previously, it has been shown that *db/db* mice develop obesity from 4 weeks of age, and hyperglycemia from 4 to 8 weeks of age (O'brien et al., 2014; Sullivan et al., 2007). In this study, we measured body weight and non-fasting glucose levels from week 14 until the animals were euthanized (week 26; Fig. S1). At 26 weeks of age, diabetic *db/db* animals displayed an increase of ∼twofold to ∼threefold in body weight and plasma glucose levels, respectively, compared to non-diabetic animals (Fig. S1). Previously, we showed that *db/db* mice also displayed increased levels of plasmatic triglyceride and glycated hemoglobin, and by week 26 they presented the main clinical signs of human DN, including a reduced nerve conduction velocity, a decreased intra-epidermal nerve fiber density and sensory loss in posterior limbs compared to non-diabetic littermates (De Gregorio et al., 2018). Thus, *db/db* mice represent a suitable T2DM model, resembling the main features of human disease.

### DRG neurons obtained from aged diabetic mice survive and regenerate in the absence of serum and neurotrophins

Although most protocols and studies regarding DRG neurons have used embryonic, neonatal or juvenile (6-8 weeks old) rodents (Katzenell et al., 2017; Liu et al., 2013; Malin et al., 2007; Owen and Egerton, 2012; Sleigh et al., 2016), other research has shown evidence that DRG neurons obtained from aged rodents are able to survive and regenerate when they are cultured *in vitro* (Fukuda and Kameyama, 1979; Fukuda, 1985; Unsicker et al., 1985). However, there is little information about whether DRG neurons of aged (≥6-month-old) diabetic mice survive and regenerate when they are cultured *in vitro* in the absence of serum and neurotrophins. Here, we optimize a protocol for culturing lumbar DRG neurons obtained from aged (6-month-old) diabetic mice. After dissociation, DRG neuronal and non-neuronal cells were attached to poly-lysine substrate for 3 h in a FBS containing medium; after the attachment, FBS was withdrawn, and cells were maintained with N2 as the unique supplement and with cytosine arabinoside (AraC) to avoid non-neuronal cell proliferation. Then, the temporal course of neurite regeneration after 24, 48, and 72 h of *in vitro* culture was analyzed. Through bright-field microscopy, the regenerative potential for both diabetic and non-diabetic-derived neuron cultures was assessed. In Fig. S2, it is possible to differentiate neuronal cells from non-neuronal cells by their bright round morphology, which extends thin neurites to branch out over time until they form incipient neurite networks after 72 h of culture. Although a mitosis inhibitor was used (AraC), it is possible to identify some non-neuronal cells in both neuronal cultures. Previously, it has been described that the effect of anti-mitotic agents start occurring after 24 h, and the maximum effect is reached after 72 h in culture, suggesting that AraC supplementation is partially useful for short-term neuronal cultures (Liu et al., 2013).

### Cultures of sensory neurons from diabetic mice displayed a reduced population of neurons and decreased neuritic regeneration

**Fig. 1.**
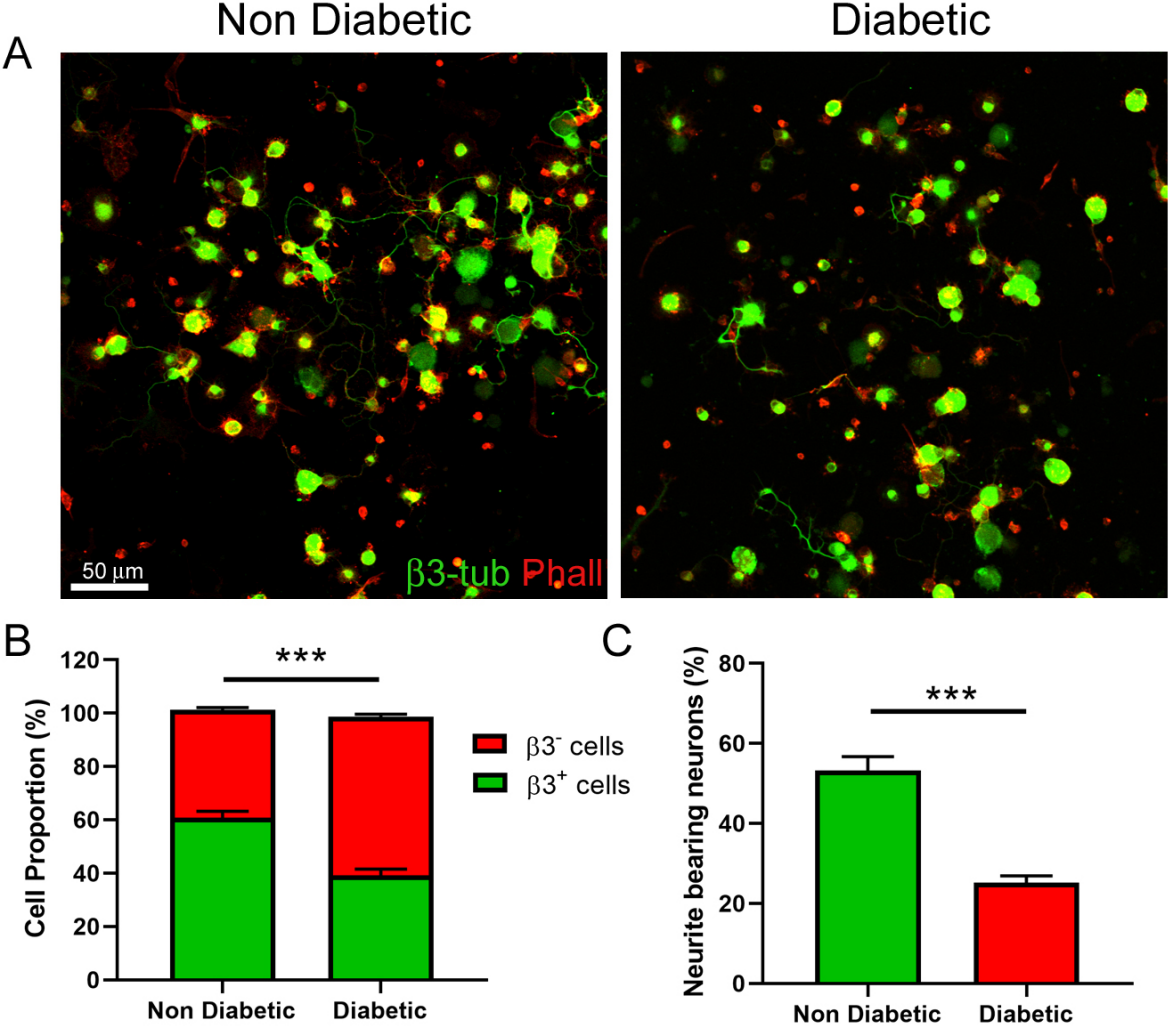
**Cultures of sensory neurons obtained from diabetic mice displayed a decreased population of neurons and neurite-bearing neurons.** (A) Representative confocal images of DRG cultures obtained from diabetic and non-diabetic mice after 48 h of culturing, stained with the pan-neuronal marker β3-tubulin (green) and the actin stain Phalloidin (red). Scale bar: 50 µm. (B) Quantification of neuronal populations in the DRG cultures obtained from diabetic and non-diabetic mice, determined as the percentage of β3-positive cells. Total cell number was assessed by Phalloidin and DAPI staining (image not shown). (C) Quantification of neurite-bearing neurons in DRG cultures obtained from diabetic and non-diabetic mice, which was determined as the percentage of β3^+^ cells extending a neurite 20 µm or more. Data are mean±s.e.m. ***P<0.001, *n*=4 (unpaired two-tailed Student's *t*-test).

Previously, DN has been associated with the apoptosis of DRG neurons and with a decreased axonal regeneration *in vivo* (Sango et al., 2017; Schmeichel et al., 2003; Srinivasan et al., 2000). Furthermore, sensory neurons derived from T1DM-adult rodents also displayed neuritic regeneration defects when cultured *in vitro* (Sakaue et al., 2003; Zherebitskaya et al., 2009). Thus, we evaluated whether cultures of DRG cells obtained from T2DM mice displayed a reduced neuronal population and a decreased capacity to extend neurites *in vitro*, compared to DRG cells obtained from non-diabetic littermates. After 48 h in culture, cells were fixed and neurons were immunostained against β3-tubulin, and the total cell population was co-stained with the actin and nuclear markers Phalloidin and DAPI, respectively. Our results showed that cultures obtained from diabetic mice presented a reduced proportion of β3-positive cells (Fig. 1A,B); meanwhile, the total cell number was not significantly decreased (non-diabetic, 72±6 cells per field; diabetic: 66±7 cells per field; *P*=0.256, Student's *t*-test, *n*=4). Furthermore, DRG neurons from diabetic mice displayed a significant reduction in neuritogenic potential, defined as the percentage of neurons extending at least one neurite (more than 20 µm) (Fig. 1C).

**Fig. 2.**
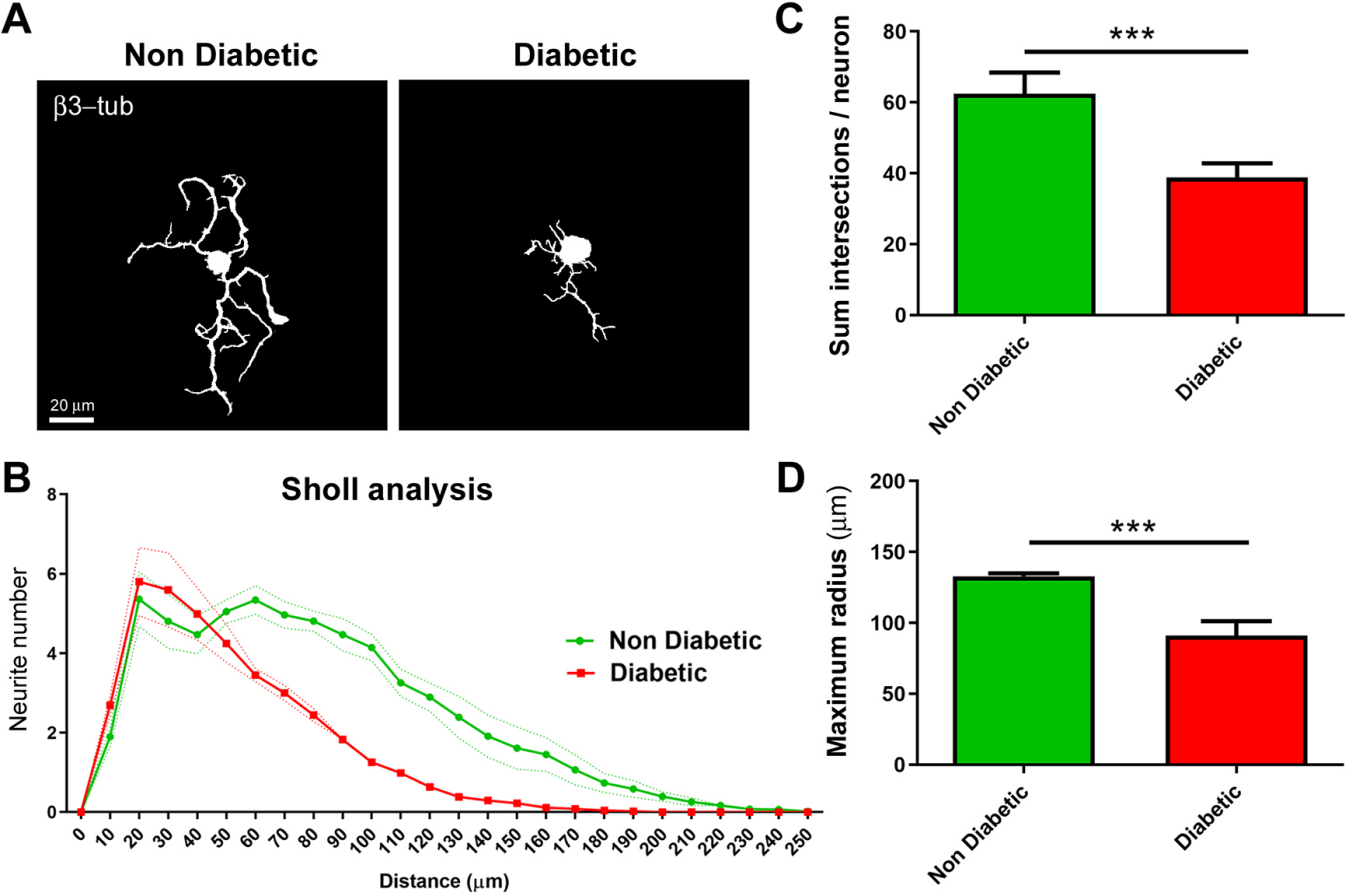
**Sensory neurons obtained from diabetic mice exhibited neurite outgrowth and neurite branching defects *in vitro*.** (A) Representative binary images of DRG neurons obtained from diabetic and non-diabetic mice after 48 h of culturing, stained with the pan-neuronal marker β3-tubulin. (B) Sholl analysis of DRG neurons. (C-D) Quantification of the mean sum of intersections per neuron (C) and the mean maximum radius reached by neurons (D), as parameters derived from Sholl analysis. Data are mean±s.e.m. ***P<0.001, *n*=4 different cultures, 120 total neurons analyzed per experimental condition (unpaired two-tailed Student's *t*-test).

Next, we analyzed the potential of neuritic regeneration specifically in neurite-bearing neurons, to evaluate whether regenerating neurons from diabetic mice exhibited neurite outgrowth defects. For this, we fixed and immunostained neurons with the neuronal marker β3-tubulin, and cultures were analyzed by confocal microscopy. Fluorescence images were transformed to binary images and Sholl morphological analyses were performed, as described previously (Ferreira et al., 2014) (Fig. 2A,B). Our results demonstrated that DRG neurons obtained from diabetic mice exhibited a reduced neurite outgrowth and branching compared to neurons derived from non-diabetic littermates, which was quantified as the mean sum of intersections per neuron (Fig. 2B,C) and the mean maximum radius reached by a neuron (Fig. 2C,D), respectively. Both parameters were derived from Sholl analysis. Taken together, our results indicate that DRG cultures from aged *db/db* mice resemble some features of DRG neurons derived from diabetic mice *in vivo*.

### Cultures of sensory neurons from diabetic mice displayed a reduced response to neuritogenic factors

One of the main advantages of using *in vitro* DRG cultures is the possibility of evaluating the relative contribution of specific cell types to DN, by analyzing their molecular and functional responses in a controlled environment. To date, there is little knowledge regarding whether the decreased regeneration response associated with T2DM is determined by intrinsic neuronal defects, or by a deficient pro-regenerative microenvironment (e.g. dysfunctional SCs or reduced levels of neurotrophins). To address this issue, we analyzed the morphological response of DRG neurons from diabetic mice to two well-known neuritogenic molecules: the extracellular matrix protein laminin (herein referring to Laminin-1; also known as Laminin-111), and the nerve growth factor NGF.

**Fig. 3.**
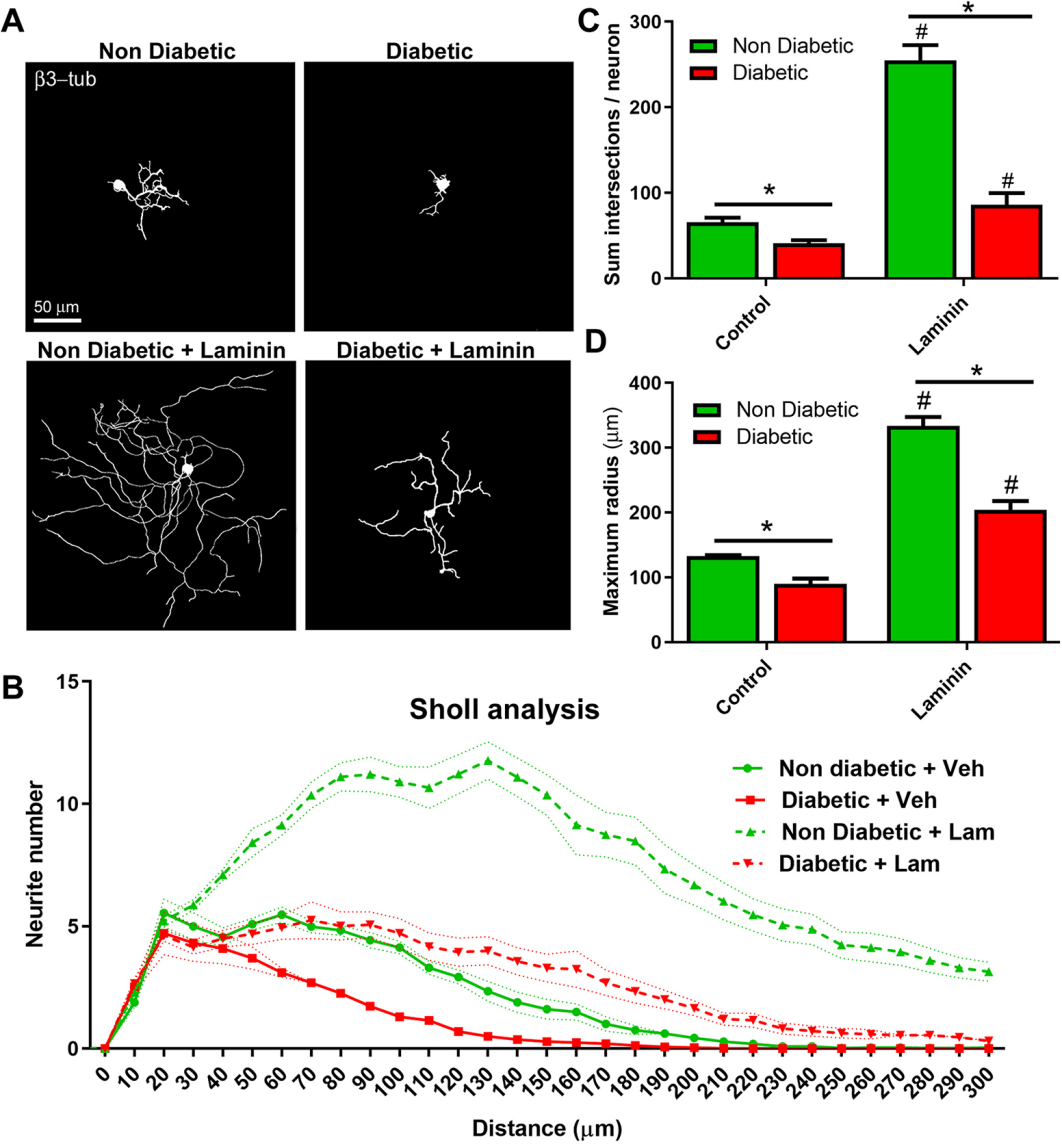
**Sensory neurons from diabetic mice displayed a reduced response to laminin *in vitro*.** (A) Representative binary images of DRG neurons obtained from diabetic and non-diabetic mice pretreated with laminin, fixed after 48 h of culturing and stained with the pan-neuronal marker β3-tubulin. (B) Sholl analysis of DRG neurons shown in A. (C,D) Quantification of the mean sum of intersections per neuron (C) and the mean maximum radius reached by each neuron (D). Data are mean±s.e.m. Asterisks and hashtags indicate significant differences as determined by two-way ANOVA with post-hoc Tukey's test; hashtags indicate significant differences with non-treated neurons (*n*=4 different cultures, 120 total neurons analyzed per experimental condition). In C, left asterisk represents *P*=0.041; right asterisk, *P*=0.0001; left hashtag, *P*=0.0001; and right hashtag, *P*=0.0316. In D, left asterisk represents *P*=0.0254; right asterisk, *P*=0.0001; and hashtags, *P*=0.0001.

**Fig. 4.**
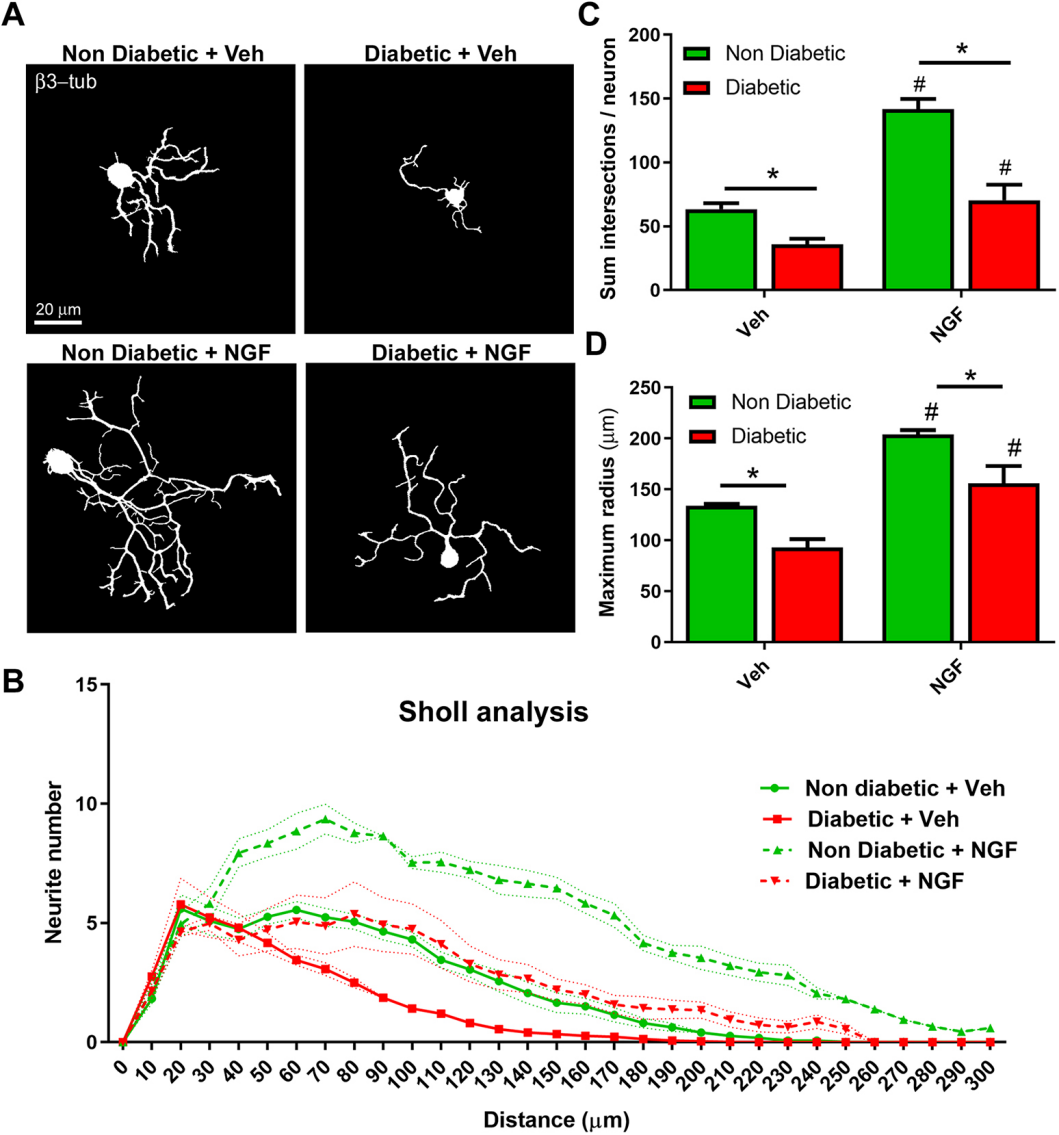
**Sensory neurons from diabetic mice displayed a partial response to NGF *in vitro*.** (A) Representative binary images of DRG neurons obtained from diabetic and non-diabetic mice treated with NGF or vehicle, fixed after 48 h of culturing and stained with the pan-neuronal marker β3-tubulin. (B) Sholl analysis of DRG neurons shown in A. (C-D) Quantification of the mean sum of intersections per neuron (C) and the mean maximum radius reached by each neuron (D). Data are mean±s.e.m. Asterisks and hashtags indicate significant differences by two-way ANOVA with post-hoc Tukey's test; hashtags indicate significant differences with non-treated neurons (*n*=4 different cultures, 120 total neurons analyzed per experimental condition). In C, left asterisk represents *P*=0.044; right asterisk, *P*=0.0001; left hashtag, *P*=0.0001; and right hashtag, *P*=0.0167. In D, left asterisk represents *P*=0.0206; right asterisk, *P*=0.0154; left hashtag, *P*=0.0004; and right hashtag, *P*=0.012.

We analyzed the morphological response of DRG neurons to laminin through a Sholl analysis (Fig. 3A,B). This analysis showed that sensory neurons from diabetic mice displayed an increased maximum radius and neurite branching compared to non-treated neurons from diabetic mice in response to laminin (Fig. 3C,D). However, this response was reduced compared to laminin-treated sensory neurons from non-diabetic mice. Similarly, sensory neurons from diabetic mice treated with NGF exhibited a significant increase in maximum radius and neurite branching compared to vehicle-treated neurons from diabetic mice Fig. 4A-D, but this treatment was not sufficient to match the morphology of NGF-treated neurons derived from non-diabetic mice. Taken together, these results suggest that neurons from diabetic mice maintain their ability to respond to external neuritogenic factors, but they may have intrinsic defects that prevent them from fully recovering the morphology of a healthy neuron.

### Culture of sensory neurons from diabetic mice as an *in vitro* model for the evaluation of experimental therapies for DN

**Fig. 5.**
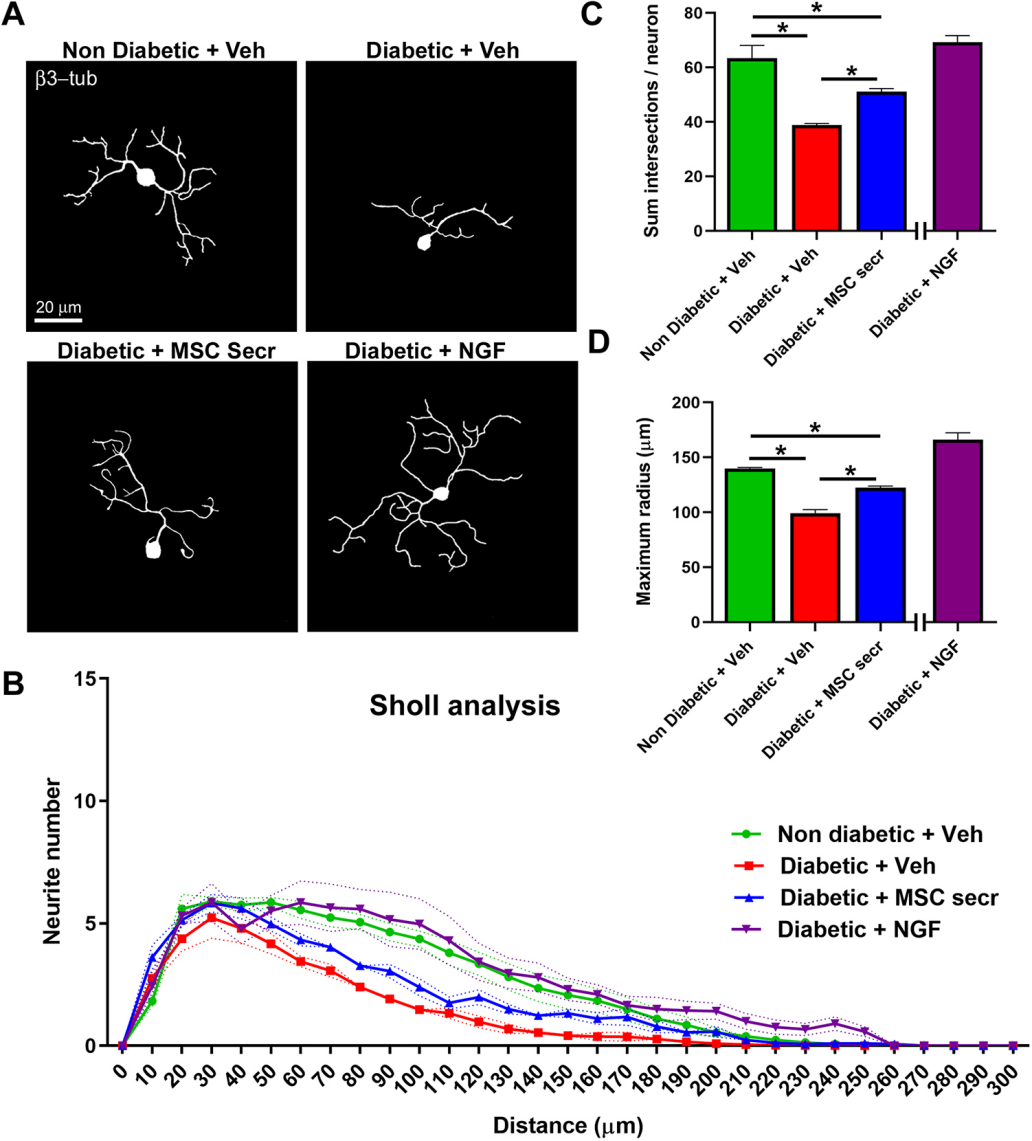
**MSC secretome treatment partially restored neurite growth and neurite branching in sensory neurons from diabetic mice.** (A) Representative binary images of DRG neurons obtained from diabetic and non-diabetic mice treated with MSC secretome or vehicle, fixed after 48 h of culturing and stained with the pan-neuronal marker β3-tubulin. (B) Sholl analysis of DRG neurons shown in A. (C-D) Quantification of the mean sum of intersections per neuron (C) and the mean maximum radius reached by each neuron (D). Data are mean±s.e.m. Asterisks indicate significant differences by one-way ANOVA with post-hoc Tukey's test (*n*=4 different cultures, 120 total neurons analyzed per experimental condition). In C, upper asterisk represents *P*=0.0249; middle asterisk, *P*=0.0001; and lower asterisk, *P*=0.0249; In D, upper asterisk represents *P*=0.0006; middle asterisk, *P*=0.0001; and lower asterisk, *P*=0.019.

Finally, we used this model as a proof of concept, to study a promising experimental therapy based on the use of the MSC secretome, which we previously evaluated in the culture of hyperglycemia-treated embryonic DRG neurons (Oses et al., 2017) and in diabetic *db/db* mice *in vivo* (De Gregorio et al., 2020). We cultured adipose tissue-derived MSCs, as described previously (Oses et al., 2017), to obtain concentrated fractions of their secretome. Next, we treated DRG neurons from diabetic and non-diabetic mice with the MSC secretome *in vitro* and carried out a morphological assessment by confocal microscopy. Sholl analysis results (Fig. 5A,B) indicated that MSC secretome-treated neurons from diabetic mice displayed a slight but significant increase in maximum radius and Sholl intersections compared to vehicle-treated neurons from diabetic mice (Fig. 5C,D), suggesting that proliferative and neurotrophic molecules present in the secretome may act directly over neurons. This result indicates that cultures of DRG neurons derived from diabetic mice constitute a suitable model to study DN mechanisms, or to assay new therapies aiming to avoid neuropathy progression.

## DISCUSSION

Although *in vivo* models of diabetes provide a physiological context highly valuable for studying DN, this system complexity has important limitations for dissecting the molecular events triggering nerve dysfunction. Thus, *in vitro* cultures have emerged as useful simplified models that complement studies carried out on whole animals. As this model allows the study of neurons in an autonomous manner, it could also be useful for complementary studies carried out *in vivo*, to demonstrate that experimental treatments that reduce the progression of DN could act directly over the neuronal cells. However, currently characterized models used for DN research are limiting, as they do not recreate the chronic and systemic damage suffered by peripheral neurons and nerves typically observed in T2DM.

In this study, we characterized the culture of DRG neurons obtained from 6-month-old diabetic animals using a morphological analysis as an easy-to-analyze readout of neuronal function. The main advantage of this model is that neurons have a chronic exposure to a harmful environment that goes beyond hyperglycemia, and that includes nerve inflammation, increased oxidative stress, dyslipidemia, insulin resistance and hypertension (Burke et al., 2017; Campero et al., 2015; De Gregorio et al., 2018). In fact, we demonstrated that sensory neurons of diabetic animals cultured in basal conditions exhibited morphological defects compared to neurons from non-diabetic mice, which could allow the analysis of other neuronal functions without adding additional toxic factors to the culture plate.

One limitation of DRG cultures is that neuronal isolation requires axotomy of both central and peripheral nerves, which activates a potent transcriptional network associated with axonal regeneration (He and Jin, 2016), and needs to be considered when studying associated signaling pathways. A second limitation is that DRG cultures include non-neuronal contaminating cells, such as fibroblasts, satellite glia, and SCs. Although it is possible to obtain highly pure DRG cultures by using mitosis blocking agents, this strategy has only been effective in long-term cultures (Hattangady and Rajadhyaksha, 2009; Liu et al., 2013).

To date, there is ample evidence that axonal regeneration is impaired in diabetes *in vivo*, but its precise mechanisms have not been completely elucidated. Among the proposed mechanisms are SC dysfunction, alteration and glycation of extracellular matrix components, reduced expression of neurotrophic factors and enhanced activity of growth-inhibitory molecules (Sango et al., 2017). Axonal regeneration is also impaired in neurons derived from diabetic animals in culture, in which molecules such as metalloproteinases (MMP-2; Ali et al., 2014), neurotrophins (neuritin; Karamoysoyli et al., 2008) and phosphatases (PTEN; Singh et al., 2014) have been implicated, but the molecular mechanisms are still poorly understood. Furthermore, the contribution of specific cell types to the causality and progression of neuropathies remains unexplored. Previous studies have shown that neurons from diabetic animals exhibited reduced levels of NGF, insulin and insulin-like signaling (Aghanoori et al., 2019; Lu et al., 2020; Peeraer et al., 2011; Tosaki et al., 2008). However, it is not clear whether these defects resulted from low levels of trophic support (e.g. by a reduced SC population or by low levels of neurotrophins secretion), or a reduced ability of neurons to respond to stimuli. Our results demonstrated that neurons from diabetic mice displayed a significant response to NFG, but it was reduced compared to neurons from non-diabetic mice treated with NGF. Previously, neurons from diabetic animals were shown to have a poor growth cone response to NGF, which could suggest signaling defects in response to this growth factor (Lu et al., 2020). On the other hand, basement membranes (BMs) play a key role in nervous system development and in maintaining neuronal function in adulthood (Yurchenco, 2011). Laminins, a major component of BMs, are enriched in peripheral nerves after injury and have been proposed as a potent stimulator of nerve regeneration (David et al., 1995; Gonzalez-Perez et al., 2013; Nieuwenhuis et al., 2018). Various studies have demonstrated that DN induces changes in BM composition, leading to an increase in the thickness and rigidity (Hill, 2009; King et al., 1989; Kundalić et al., 2014; To et al., 2013); however, these structural changes have been poorly explored. Although laminin level seems to be similar between nerves from healthy and diabetic patients (Hill, 2009; To et al., 2013), several studies indicated that neurons from diabetic animals displayed a reduced response to laminin, with an effect of reduced adhesion to extracellular matrix proteins and a decreased laminin quality triggered by excessive glycation (Duran-Jimenez et al., 2009; Federoff et al., 1993; King, 2001; Sango et al., 1995). Here, we demonstrated that DRG neurons from diabetic mice exhibited a reduced regenerative response to laminin, and this response was intrinsic to neurons as the laminin coating was the same in both conditions. Thus, it is possible that regenerative defects associated with neurons of diabetic mice *in vivo* could be determined by both intrinsic defects (e.g. by a reduced adhesion or signaling defects) and poor quality or decreased levels of pro-regenerative extracellular matrix proteins and neurotrophins.

Finally, we used this *in vitro* model to evaluate the efficiency of a new therapy based on the MSC secretome. In a previous study, we showed that the systemic administration of the MSC secretome to *db/db* mice was able to completely reverse the main structural and functional defects associated with DN (De Gregorio et al., 2020); however, it was not possible to evaluate whether the MSC secretome acts directly over neurons (in a cell-autonomous manner), or acts indirectly by improving the neuronal microenvironment. Here, we demonstrated that the MSC secretome partially reverts the impaired regeneration of sensorial neurons from diabetic animals, suggesting that the MSC-derived secretome could act directly over neurons, and indirectly ameliorating the noxious nerve microenvironment. As the MSC secretome is composed of hundreds of different molecules, it may be difficult to identify those that are responsible for the neuritogenic activity. We previously carried out a proteomic analysis of the MSC secretome and detected the neuron-derived neurotrophic factor (NDNF) as one of the most abundant proteins (De Gregorio et al., 2020). NDNF has been previously described as a neurotrophic factor that induces neuritic outgrowth and exerts neuroprotection against harmful stimuli in mouse hippocampal neurons *in vitro* (Kuang et al., 2010). Thus, it remains to be determined whether this molecule is the main molecule responsible for the neuritogenic effect of the MSC secretome. Previously, it was shown that brain-derived neurotrophic factor (BDNF) mediated axonal outgrowth in cortical and hippocampal neurons treated with MSC secretome *in vitro* (Martins et al., 2017). Thus, the neuritogenic effect could be due to a set of molecules rather than a single one.

The currently characterized *in vitro* model could also be extrapolated for studying other parameters of interest associated with T2DM, such as cell survival, synaptic structure formation and activity, electrophysiological responses, interactions with glial cells, etc., in a controlled environment, either under standard or experimental conditions of interest.

## MATERIALS AND METHODS

### Animals

Transgenic mice (BKS.Cg-m^+^/^+^Lepr^db^/J) that spontaneously develop T2DM were purchased from The Jackson Laboratory. Female diabetic (db/db) and non-diabetic (db/^+^) mice were housed at constant temperature and humidity, with a 12-h light/dark cycle and unrestricted access to standard chow and water. All animal protocols were approved by the Animal Ethics Committee of Universidad del Desarrollo. Six-month-old mice were euthanized by an overdose of anesthesia, and L3-L5 ganglia were dissected and used for culture preparation (additional ganglia or all the ganglia may be used, according to work objectives).

### Measurement of blood glucose

Non-fasting blood glucose levels were measured using a glucometer (Accu-Chek Performa System, Roche). Blood samples were obtained from the tail of alert animals every 2 weeks (from week 14 to week 26). Mice with a sustained glycemia value of over 250 mg/dl were considered diabetic.

### Isolation and culturing of adipose-derived MSCs

Human adipose-derived MSCs were isolated from fresh subcutaneous adipose tissue samples (abdominal region) obtained from liposuction aspirates of four healthy female human donors undergoing cosmetic liposuction at Clínica Alemana, Chile, as described previously (Oses et al., 2017). Written informed consent was obtained for all samples, and the protocols used were approved by the Ethics Committee of Universidad del Desarrollo and were carried out according to the ethical principles outlined in the Declaration of Helsinki. MSC culture and cell characterization have been described previously (Oses et al., 2017). Briefly, when cells reached 70% confluency, plates were rinsed three times with PBS and cells were incubated for 48 h in α-minimum essential medium without fetal bovine serum (FBS). The media were centrifuged at 400 ***g*** for 10 min to remove whole cells, and the supernatants were centrifuged again at 5000 ***g*** for 20 min to remove cell debris. Finally, conditioned media were filtered in 0.22-µm filters, concentrated 10× (v/v) using 3 kDa cut-off filters (Millipore) and stored at −80°C until use.

### Primary cultures of DRG neurons

DRGs from L3-L5 were dissected from 26-week-old animals, and enzymatically digested by incubation for 25 min at 37°C with 0.6% trypsin (Gibco), and then for 25 min at 37°C with 0.5% collagenase type I (Gibco). The enzymatic activity was halted by adding 1 ml of FBS, and tissue suspension was centrifuged at 400 ***g*** for 5 min. Then, the pellet was resuspended in Dulbecco's modified Eagle's medium (DMEM)/F12 (Gibco) with 10% FBS and mechanically dissociated. A total of 5000 cells were plated on coverslips coated with 0.05% poly-D-lysine (Sigma-Aldrich) or Poly-D-lysine plus laminin (1 µg/µl, Sigma-Aldrich). Neurons were allowed to attach to the substratum for 4 h before changing medium to DMEM/F12 containing N2 supplement (Gibco) and AraC (Sigma-Aldrich). Where indicated, NGF (10 ng/ml, Alomone Labs), MSC secretome (containing 5 µg/ml of protein) or vehicle (PBS) were added to the culture medium and replaced after 24 h of treatment. After 44 h in culture (48 h in total), cells were fixed with 4% paraformaldehyde containing 4% sucrose for 20 min, and neurons were immunostained with β3-tubuline antibody (TU-20, 1:150, Santa Cruz Biotechnology). Phalloidin (Santa Cruz Biotechnology, sc-363794, 1:500) and DAPI (1:500, AppliChem) were used to stain the actin cytoskeleton and nuclei, respectively, of neuronal and non-neuronal cells. Samples were analyzed by confocal microscopy (Fluoview FV10i; Olympus). All the experiments were carried out with four different cultures per condition.

### Sholl analysis

Sholl analysis was carried out as described previously (Ferreira et al., 2014). Briefly, confocal images were transformed to 8-bit binary images, and then were quantified with the Sholl analysis tool (Fiji software; National Institutes of Health). The distance/neurite-number profile, the maximum radius reached by each neuron, and the sum of intersections for each neuron were evaluated. To facilitate the analysis of neuronal morphology, we randomly selected isolated neurons. To avoid quantifying dying neurons, we analyzed only neurons extending at least one neurite 20 µm or more. Samples from four animals per experimental group were evaluated (30 neurons per experiment, 120 neurons in total).

### Statistical analysis

Quantitative data were presented as mean±s.e.m. Comparisons between two different conditions were analyzed using an unpaired two-tailed Student's *t*-test. Comparisons between groups were performed using one-way ANOVA or two-way ANOVA with Tukey's post-test. Grouped repeated measures were analyzed with two-way ANOVA with Bonferroni post-test. *P*<0.05 was considered statistically significant.

## Supplementary Material

Supplementary information
