# Supplementary material for: Sensory neuron cultures derived from adult db/db mice as a simplified model to study type-2 diabetes-associated axonal regeneration defects
Source: Dis Model Mech. 2021 Jan 22;14(1):dmm046334. doi: 10.1242/dmm.046334 (PMC7847260; doi:10.1242/dmm.046334)
Supplement: Supplementary information [file dmm-14-046334-s1.pdf]

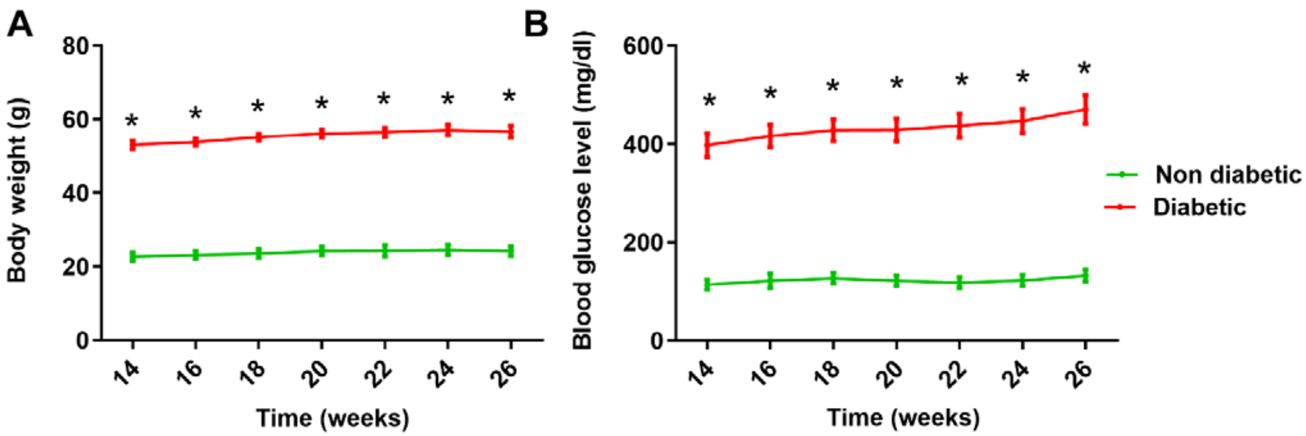

Figure S1: Diabetic *db/db* mice displayed an increased body weight and blood glucose level compared to non-diabetic animals. (A-B) Time course of body weight (A) and non-fasting blood glucose levels (B) in diabetic and non-diabetic mice. Data are presented as mean ± S.E.M. Asterisks represent significant differences (two-way ANOVA and Bonferroni post-test, n=8 animals per experimental condition).

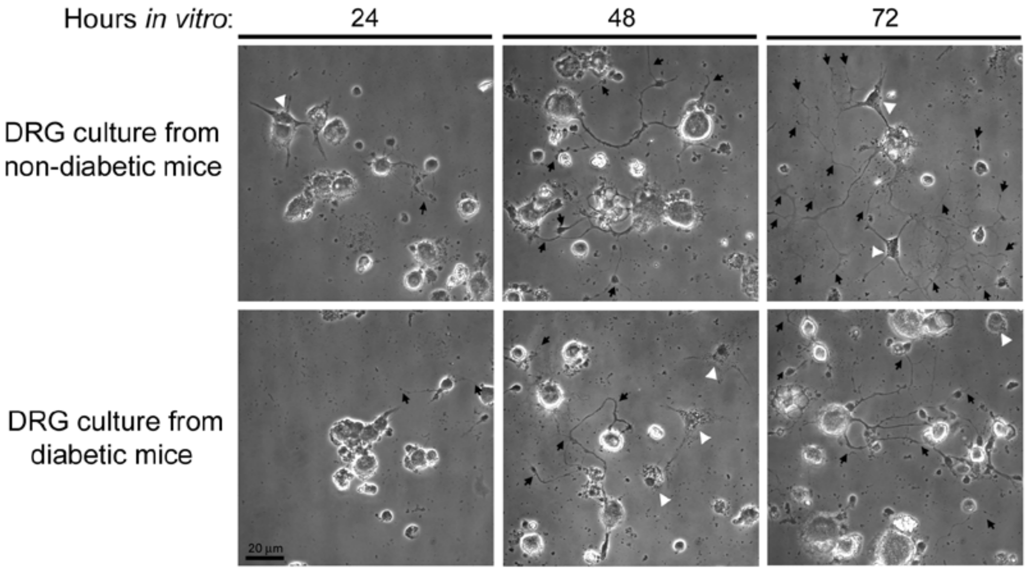

Figure S2: DRG neurons derived from 6-month-old diabetic mice are able to survive and spread neurites in absence of serum and neurotrophins. Representative bright field images of DRG neurons obtained from diabetic and non-diabetic mice after 24, 48, and 72 hours of *in vitro* culture. Black arrows indicate regenerating neurites and white arrowheads indicate the presence of evident non-neuronal cells. Bar represents 20 µm (representative of four different cultures per experimental condition).

Disease Models & Mechanisms • Supplementary information
